# DNA Methylation Patterns in the Hypothalamus of Female Pubertal Goats

**DOI:** 10.1371/journal.pone.0165327

**Published:** 2016-10-27

**Authors:** Chen Yang, Jing Ye, Xiumei Li, Xiaoxiao Gao, Kaifa Zhang, Lei Luo, Jianping Ding, Yunhai Zhang, Yunsheng Li, Hongguo Cao, Yinghui Ling, Xiaorong Zhang, Ya Liu, Fugui Fang

**Affiliations:** 1 Anhui Provincial Laboratory of Animal Genetic Resources Protection and Breeding, College of Animal Science and Technology, Anhui Agricultural University, 130 Changjiang West Road, Hefei, Anhui 230036, China; 2 Anhui Provincial Laboratory for Local Livestock and Poultry Genetic Resource Conservation and Bio-Breeding, 130 Changjiang West Road, Hefei, Anhui 230036, China; 3 Department of Animal Veterinary Science, College of Animal Science and Technology, Anhui Agricultural University, 130 Changjiang West Road, Hefei, Anhui 230036, China; Harbin Institute of Technology, CHINA

## Abstract

Female pubertal development is tightly controlled by complex mechanisms, including neuroendocrine and epigenetic regulatory pathways. Specific gene expression patterns can be influenced by DNA methylation changes in the hypothalamus, which can in turn regulate timing of puberty onset. In order to understand the relationship between DNA methylation changes and gene expression patterns in the hypothalamus of pubertal goats, whole-genome bisulfite sequencing and RNA-sequencing analyses were carried out. There was a decline in DNA methylation levels in the hypothalamus during puberty and 268 differentially methylated regions (DMR) in the genome, with differential patterns in different gene regions. There were 1049 genes identified with distinct expression patterns. High levels of DNA methylation were detected in promoters, introns and 3′-untranslated regions (UTRs). Levels of methylation decreased gradually from promoters to 5′-UTRs and increased from 5′-UTRs to introns. Methylation density analysis demonstrated that methylation level variation was consistent with the density in the promoter, exon, intron, 5′-UTRs and 3′-UTRs. Analyses of CpG island (CGI) sites showed that the enriched gene contents were gene bodies, intergenic regions and introns, and these CGI sites were hypermethylated. Our study demonstrated that DNA methylation changes may influence gene expression profiles in the hypothalamus of goats during the onset of puberty, which may provide new insights into the mechanisms involved in pubertal onset.

## Introduction

The hypothalamus-pituitary-gonadal (HPG) axis plays a critical role in the onset of puberty. Gonadotropin-releasing hormone (GnRH) is secreted by neurosecretory neurons located in the hypothalamus in primates and in the preoptic region in rodents. GnRH secretion is inhibited in embryos and infants and later reactivated, with pulsatile secretion before the onset of puberty [1, 2].

A complex network of genes is responsible for the control of puberty[1, 3–5], and epigenetic regulation has emerged as playing an important role in the regulation of puberty onset in recent years. Several studies have indicated that the reactivation of GnRH secretion with pubertal initiation may be associated with downregulation of the Makorin ring finger 3 gene (*MKRN3*)[2, 3, 6–8]. Kisspeptin, a GnRH agonist encoded by the *KISS1* gene, also plays an essential role in regulating the timing of puberty[9–11]. In female rodents, kisspeptin neurons in the anteroventral periventricular nucleus are critical for GnRH positive feedback regulation[12–14]. In addition, monogenic mutations in several genes, including *KISS1*, *MKRN*, *TAC3* (tachykinin 3), and *GPR54* (G-protein-coupled receptor 54) have also been associated with pubertal initiation failure[9, 10, 15–17]. Furthermore, several central nodes have been identified in previous reports, including the zinc finger–containing gene *EAP1* (enhanced at puberty 1), the POU-domain gene *Oct2* and the homeodomain gene *TFT1*[1, 5].

While there have been a number of reports describing epigenetic changes associated with differential gene patterns and the regulation of pubertal onset, the present study provides the first whole-genome DNA methylation analysis during puberty. In order to understand the methylation patterns in the hypothalamus during puberty, we performed whole-genome bisulfite sequencing (WGBS) and RNA-sequencing (RNA-Seq) to determine DNA methylation and gene expression changes during puberty in goats. Our results demonstrate very low global DNA methylation in prepubertal and pubertal stages which both showed positive and negative correlations with gene expression patterns in the goat hypothalamus.

## Materials and Methods

### Ethics Statement

All goats in this study were housed in open sheepfolds and fed ad libitum. The sacrifice of goats used sodium barbital after anesthesia. All procedures involving animals were approved by the Animal Care and Use Committee of Anhui Agricultural University.

### Sample collection and preparation

Three pubertal Anhuai goats aged 4.5–5 months, weighing 17.43 ± 1.63 kg, were used in this study. Animals were monitored daily by observing vaginal physiological changes and rams test conditions[18]. Rams test conditions were performed twice at 08:00 and 16:00 hours. The cunnus of pubertal goats was inflamed, with histological observation of some mature follicles in the ovaries. Three prepubertal goats (aged 2.5 months, weighing 9.60 ± 2.36 kg) were sacrificed after anesthesia with 0.1 ml xylazine hydrochloride injection (Muhua China, Lot number 150804). Hypothalami were collected and stored at −80°C until use. DNA was extracted using an AxyPrep™ Multisource Genomic DNA Miniprep Kit (Corning, APMNMSGDNA-50) and purity checked using a NanoPhotometer® spectrophotometer (Implen, West Lake Village, CA, USA) and agarose gel electrophoresis. The genomic DNA was fragmented by sonication and bisulfite modification used the EZ DNA Methylation-Gold™ Kit (Zymo Research, D5005 & D5006). RNA was extracted using an E.Z.N.A.® Total RNA Kit II (Omega Bio-tek, R6934-01) and purity was checked using the NanoPhotometer® spectrophotometer. RNA concentration was measured with a Qubit® RNA Assay Kit in a Qubit® 2.0 Fluorometer (Life Technologies, San Francisco, CA, USA).

### Whole-genome bisulfite sequencing library preparation, quantification and sequencing

Genomic DNA (5.2 μg), spiked with 26 ng λ DNA, was fragmented by sonication to 200–300 bp with a S220 focused-ultrasonicator (Covaris, Woburn, MA, USA), which was followed by adenylation and end-repair. Cytosine-methylated barcodes were ligated to sonicated DNA and these DNA fragments were treated with an EZ DNA Methylation-Gold™ Kit (Zymo Research, D5005 & D5006) to achieve single-stranded DNA fragments, which were then amplified by PCR using KAPA HiFi HotStart Uracil+ ReadyMix (Kapa Biosystems, Wilmington, MA, USA) according to the manufacturer’s instructions. Library concentration was quantified by quantitative PCR (Life Technologies, San Francisco, CA, USA), and insert size was inspected using an Agilent Bioanalyzer 2100 system (Agilent Technologies, Santa Clara, CA, USA). DNA methylation analysis was performed using a HiSeq 2500 platform (Illumina, San Diego, CA, USA) according to the manufacturer’s instructions.

### DNA methylation data analysis

The bisulfite conversion rate as determined by the analysis of λ DNA was ≥99.55% and the rate of reads mapping to the reference genome was ≥77%. To identify methylation sites, we performed a sliding-window approach that is conceptually similar to approaches used for bulk bisulfite sequencing (http://www.bioconductor.org/packages/2.13/bioc/html/bsseq.html), with a step size of 600 bp and window size w = 3,000 bp[19]; the sum of unmethylated and methylated reads counts were calculated in each window. The swDMR software (http://122.228.158.106/swDMR/) that uses a sliding-window approach was used to identify differentially methylated regions. The Fisher test was implemented for detecting the DMRs.

### RNA-Seq library preparation, quantification and sequencing

Total RNA (3 μg per sample) was used for sample preparation. Ribosomal RNA was removed with a Ribo-zero™ rRNA Removal Kit (Epicentre, Madison, WI, USA), and the rRNA free residue was cleaned up by ethanol precipitation. Secondly, using the rRNA-depleted RNA, sequencing libraries were generated by using a NEBNext® Ultra™ Directional RNA Library Prep Kit for Illumina® (New England Biolabs, Ipswich, MA, USA) according to the manufacturer’s recommendations. In order to choose 150−200 bp cDNA fragments preferentially, the AMPure XP system (Beckman Coulter, Beverly, CA, USA) was used to purify the library fragments. cDNA adaptor-ligation was performed using 3 μl USER enzyme (New England Biolabs) at 37°C for 15 min followed by 95°C for 5 min before PCR. PCR was then performed with universal PCR primers, Phusion high-fidelity DNA polymerase, and Index Primer. Finally, the purity of products was checked with an AMPure XP system (Beckman Coulter, Brea, CA, USA) and library quality evaluated using the Agilent Bioanalyzer 2100 system (Agilent Technologies). A HiSeq 2000 platform (Illumina) was used to sequence and generate 100-bp paired-end reads.

### RNA-Seq data analysis

Cuffdiff (v2.1.1) was used to calculate fragments per kilobase of exon per million reads (FPKMs) of genes in each sample[20]. In each gene group, gene FPKMs were computed by summing the FPKMs of transcripts. For biological replicates, transcripts or genes with a *P*-adjust <0.05 were assigned as differentially expressed.

### GO and KEGG enrichment analysis

Gene ontology (GO) enrichment analyses of genes related to DMRs was implemented by the GOseq R package[21]. A *P*-value <0.05 was considered statistically significant. In Kyoto Encyclopedia of Genes and Genomes (KEGG) pathways, KOBAS software[22] was used to test the statistical enrichment of DMR-related differential gene expression.

### Data availability statement

The authors state that all data necessary for confirming the conclusions presented in the article are represented fully within the article. Sequence data has been submitted to the Sequence Read Archive (SRA) and the accession number is SRP077789.

## Results

### Methylation profiles during puberty

Global DNA methylation analysis of the hypothalamus was performed with 30× genome coverage and >99% conversion efficiency by WGBS. Between prepubertal and pubertal stages of development, >18,000,000 methylcytosines (mCs) were detected, and the average percentage of mC methylation estimated in the whole genome was 1.91% in prepuberty and 1.87% in puberty (S1 Table). In line with previous reports[23], the percentage of methylated CpG dinucleotides (mCG) exceeded 98% and there was only 2% non-CpG methylation (mCHG) from prepuberty to puberty (S1 Fig). There was 0.5–0.9 kb/bin prepubertal and pubertal methylation, with hypermethylated cytosines (S2 Fig). Analyses of methylation profiles of each chromosome demonstrated more mCG (0.6–0.8 kb/bin), compared with <0.01 kb/bin mCHG and mCHH (H = adenine, thymine, or cytosine; S2 Fig).

### DNA methylation in CpG islands

**Fig 1:**
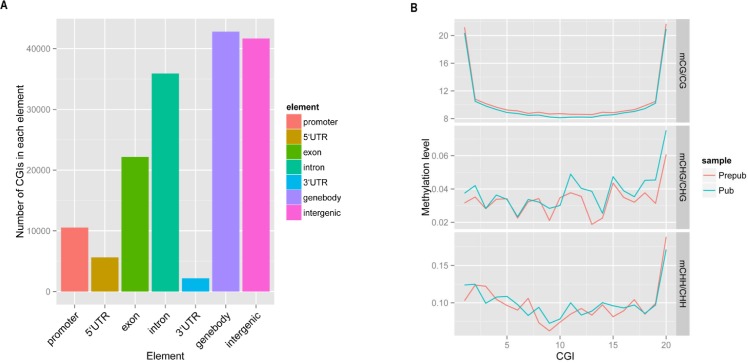
Analysis of CpG islands. In CGI analysis, every CGI was divided into 20 bin for evaluating the methylation level. (A) Distribution of CGI sites in different gene elements. (B) Different methylation levels of cytosine (percentage) between prepubertal and pubertal stages. Prepub = prepuberty; Pub = puberty; CGI = CpG island.

As indicated by previous studies, DNA methylation predominantly occurs in CGI sites, which is consistent in the current study. We investigated the distribution of CGI in different gene elements, and found the most enriched elements were gene bodies, exons, introns and intergenic regions (Fig 1A), with hypermethylated CGI sites in both prepubertal and pubertal stages of development (S1 and S2 Files). From the prepubertal stage to puberty, the methylation level in CGI sites decreased in CG sites, coinciding with the global methylation pattern in the hypothalamus (Fig 1B, S3 File). Interestingly, the hypermethylated C sites were located at the edge of CGI sites. Compared with mCG, mCHG and mCHH showed fluctuating profiles, and both of them increased at 20 bin of CGI.

### Methylation patterns in different gene contents

**Fig 2:**
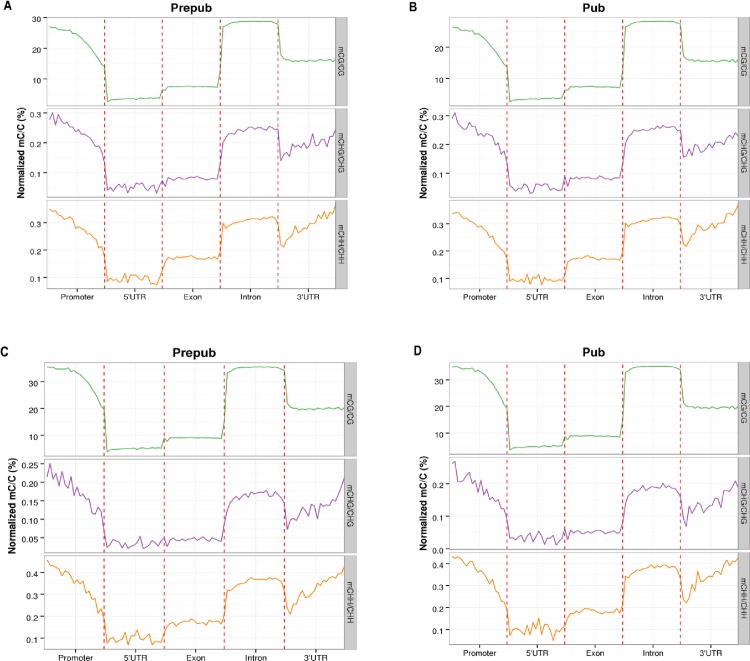
Methylation level and density in different gene elements during prepubertal and pubertal stages. Prepub = prepuberty; Pub = puberty.

DNA methylation has various functions, and methylation can occur in different locations. We evaluated the methylation level affecting different functions of genes. In promoters (the region from 2 kb upstream of the transcription start site), introns and 3′-UTRs, the level of methylation was higher than that in 5′-UTRs and exons during puberty (Fig 2A and 2B). Levels of methylation decreased gradually from promoter to 5′-UTR in the genome, promoting gene expression. In the 5′-UTRs, exons and introns, the methylation level of C sites showed relatively stable patterns. There was stable DNA methylation in CG sites in the 3′-UTRs, but an increase from 3′-UTRs to introns in the genome with an increased trend in mCHG and mCHH. Methylation density analysis identified analogous trends to the methylation level changes from prepuberty to puberty in the gene contents (Fig 2C and 2D), indicating a close relationship between level of methylation and methylation density. Interestingly, methylation of exons was low compared with introns in the gene content (Fig 2C and 2D), which was consistent with a previous report[24]. The function of DNA methylation is usually to repress gene expression. In the gene region, the activity of transcription of exons is higher than that of introns during gene transcription. This could be the reason why the methylation level of introns is higher than that of exons.

### Different methylation regions between prepuberty and puberty

**Fig 3:**
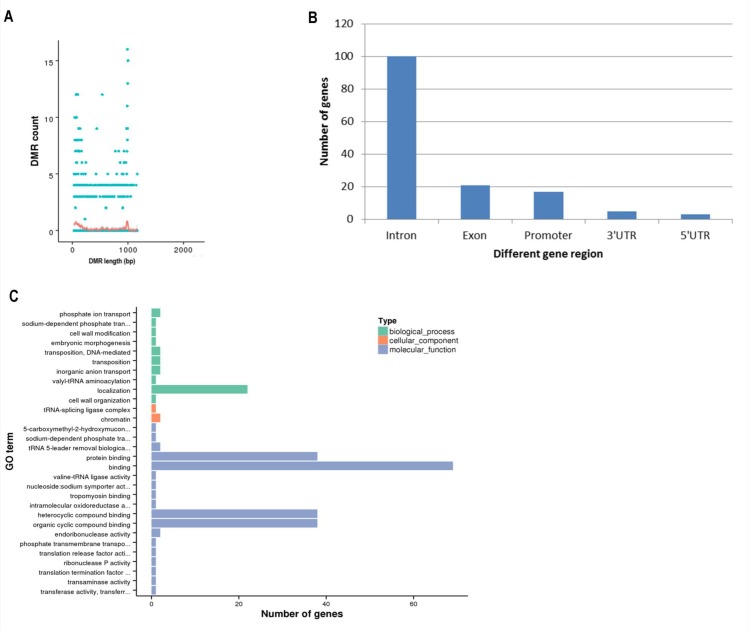
Different methylation regions in prepubertal and pubertal stages. (A) The relationship between DMR length and count. (B) Distribution of methylated genes in DMRs. (C) The pathways enriched in differentially methylated genes in DMRs, as determined by GO analysis. Prepub = prepuberty; Pub = puberty; DMR = differentially methylated region; GO = gene ontology.

Specific methylation patterns were analyzed across the whole genome during the prepubertal and pubertal stages, and 268 differentially methylated regions (DMRs) were identified. DMR length was approximately 0–1000 bp (Fig 3A, S4 File), and the methylation level in the hypothalamus was lower in puberty than that in prepuberty. Nine of these DMRs were methylated in puberty but unmethylated in prepuberty, while nine other DMRs were identified in which methylation disappeared from prepuberty to puberty. Upon analysis of these specific DMRs, two genes (*NLRC5*, *PLCXD3*) were found to be hypermethylated and five genes (*PPM1D*, *CD226*, *SMOC1*, *GRID1* and *LOC10219031*) became hypomethylated during the onset of puberty (S5 File). Interestingly, the regions displaying different methylation levels were the introns rather than the promoters (Fig 3B). To characterize genes that were detected in the DMRs, GO and KEGG pathway analyses were performed. GO analyses revealed that the differentially methylated genes were most enriched in localization, protein binding, binding, heterocyclic compound binding and organic cyclic compound binding (Fig 3C). KEGG pathway analyses identified genes involved in the oxytocin signaling pathway, estrogen signaling pathway, GnRH signaling pathway and gamma-aminobutyric acid (GABA)ergic synapse pathway, which is closely related to the timing of puberty, including *RYR1*, *ABAT*, *MAP3K4* and *FKBP52*.

### Relationship between DNA methylation and gene expression

**Fig 4:**
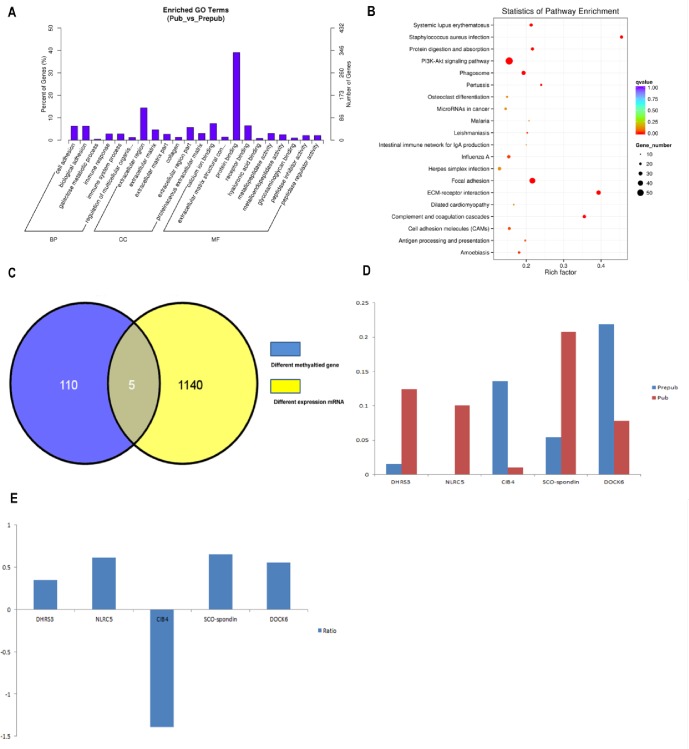
Gene expression analysis in prepubertal and pubertal stages. (A) The enriched pathways on the basis of different gene expression, as determined by GO analysis. (B) The enriched pathway on the basis of different gene expression, as determined by KEGG analysis. Rich factor refers to the ratio between the number of genes enriched in a pathway and annotated in DMR. Rich factor and enrichment are positively correlated. Q-value is the P-value that was corrected after multiple hypothesis testing. Q-value and enrichment are negatively correlated. (C) Five genes were detected, and their expression and methylation patterns were both altered during puberty onset. (D) Methylation level changes in *DHRS3*, *NLRC5*, *CIB4*, *DOCK6* and *SCO-spondin* during prepubertal and pubertal stages. (E) The ratio of mRNA between prepubertal and pubertal stages. A positive ratio represents upregulation and a negative ratio represents downregulation. Prepub = prepuberty; Pub = puberty; BP = biological process; CC = cellular component; MR = molecular function; GO = gene ontology; KEGG = Kyoto Encyclopedia of Genes and Genomes; DMR = differentially methylated regions.

To investigate the relationship between DNA methylation changes and gene expression, RNA-Seq analysis was performed to study the changes in gene expression during puberty. A total of 1048 differentially expressed genes were detected, with 21.95% downregulation (all others were upregulated, S6 File). Through GO and KEGG pathway analyses, these genes were found to be enriched in pathways such as the protein binding pathway, extracellular matrix pathway and receptor binding pathway (Fig 4A and 4B). Three of these genes were only expressed in puberty and have been implicated in pubertal onset (*GNG13*, *GH1 and LOC102171600*) although methylation patterns of these genes did not change from prepuberty to puberty. Five genes (*DHRS3*, *NLRC5*, *CIB4*, *DOCK6* and *SCO-spondin*) that showed various methylation patterns during puberty, had altered expression levels (Fig 4C). Interestingly, *DHRS3*, *NLRC5*, *CIB4* and *SCO-spondin* showed a positive correlation between DNA methylation and gene expression, and *DOCK6* showed a negative correlation (Fig 4D and 4E).

## Discussion

In the present study, the genome-wide DNA methylation of the goat hypothalamus during the prepubertal and pubertal stages of development was analyzed for the first time. Through WGBS, 268 DMR and 117 differentially methylated genes were found in the hypothalamus genome, and we identified different DNA methylation patterns from prepuberty to puberty. Two genes, *GnRH* and *KISS1*, which play important roles in the onset of puberty[9–11], showed no changes in methylation patterns in the present study. A differential methylation profile of the *KISS1* promoter has previously been demonstrated[25], and previous studies have indicated that the methylation of CpG sites in the promoter of the gene body of the *GnRH* gene showed decreased patterns across puberty[26, 27]. The reason for the differences in methylation variation of *KISS1* and *GnRH* compared with the results in the present study may be that our study analyzed the global DNA methylation patterns of genes, rather than promoter or gene body DNA methylation patterns. In addition, our study found that the methylated region of genes was mostly intronic instead of in the promoter, which has been the focus of several previous reports, perhaps because of the effect of enrichment of CpG on transcription[25, 28, 29]. However, the function of methylated introns is not well understood.

Several genes were methylated (*NLRC5*, *PLCXD3*) and some genes were demethylated (*PPM1D*, *CD226*, *SMOC1*, *GRID1*, *LOC102190311*), from prepuberty to puberty. These genes may play important roles in the pathways that may be associated with the onset of puberty, although further studies are necessary to elucidate the functional roles of these genes during puberty. In vertebrates, CGI sites are usually unmethylated or display low levels of methylation[30]. Methylated CpG sites in promoter regions could repress the combination of transcription factors and promoters to inhibit the expression of genes. CpG sites can regulate the effect of transcription via changing methylation states[31–33]. In the current study, analyses of CGI sites demonstrated that the gene regions whose methylation level changed from prepuberty to puberty were gene bodies, intergenic regions, introns and exons, rather than promoter regions, which is not consistent with previous reports[34]. These CGI sites that were not located in the promoter may exert an important effect on regulating gene expression[35]. For example, a study by Sleutels et al. found that *Air*, a noncoding RNA initiated at a CGI site within intron 2 of the *Igf2r* gene, is crucial for silencing of the paternal allele[36]. Similarly, another study reported that intron 10 of the *Kcnq1* gene was the origin of a noncoding transcript, which is necessary for imprinting of several genes in this domain[37, 38]. The majority of CGI sites were hypermethylated in the present study, indicating the low methylation level of CGI sites in prepuberty and puberty. Previous studies have shown that DNA methylation positively[39, 40] or negatively correlated[41] with gene expression. RNA-Seq revealed that different genes display differential expression patterns from prepuberty to puberty in our study, likely influenced by DNA methylation changes. Most of these genes showed different methylated regions in the gene body instead of the promoter, and genes with hypermethylation often showed higher expression. This coincided with previous studies which showed that methylation in gene bodies may have a positive effect on gene expression[40]. However, further studies are required to confirm whether differential gene expression is indeed caused by DNA methylation changes.

To our knowledge, our study presents the first genome-wide analysis of DNA methylation profiles of the goat hypothalamus during puberty, and the relationship between epigenetic changes and resultant gene expression has also been determined. These data will be informative in providing a basis for better understanding of the epigenetic regulation of pubertal onset.

## Supporting Information

S1 Fig Percentage of methylated cytosines in prepubertal and pubertal stages. (DOCX)

S2 Fig DNA methylation levels in prepuberty and puberty. (DOCX)

S1 File Methylation level of CGI sites in prepuberty. (XLSX)

S2 File Methylation level of CGI sites in puberty. (XLSX)

S3 File CGI methylation level per window in prepuberty and puberty. (XLSX)

S4 File List of DMRs. (XLS)

S5 File List of hypomethylated and hypermethylated genes. (XLSX)

S6 File List of differentially expressed genes. (XLS)

S1 Table The number and percentage of different mC contents in samples from goats in prepuberty and puberty. (DOCX)
