# Supplementary figures and images for: DNA Methylation Patterns in the Hypothalamus of Female Pubertal Goats
Source: PLoS One. 2016 Oct 27;11(10):e0165327. doi: 10.1371/journal.pone.0165327 (PMC5082945; doi:10.1371/journal.pone.0165327)

**S2.**


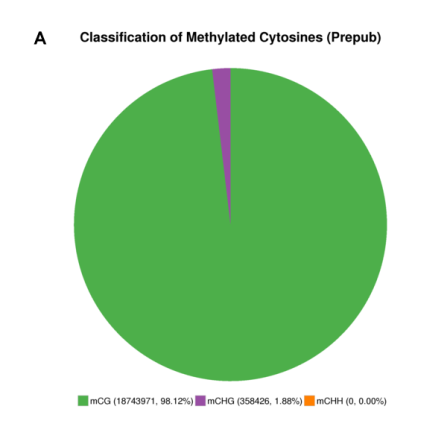


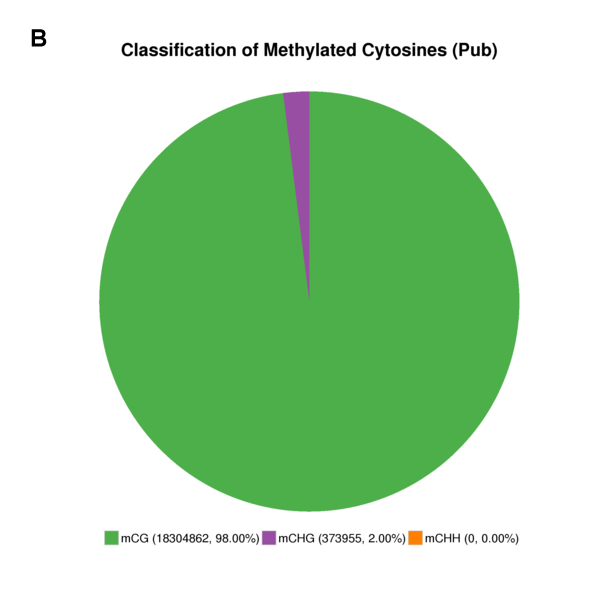

Supplement: S1 Fig — (DOCX) [file pone.0165327.s001.docx]

**S3.**


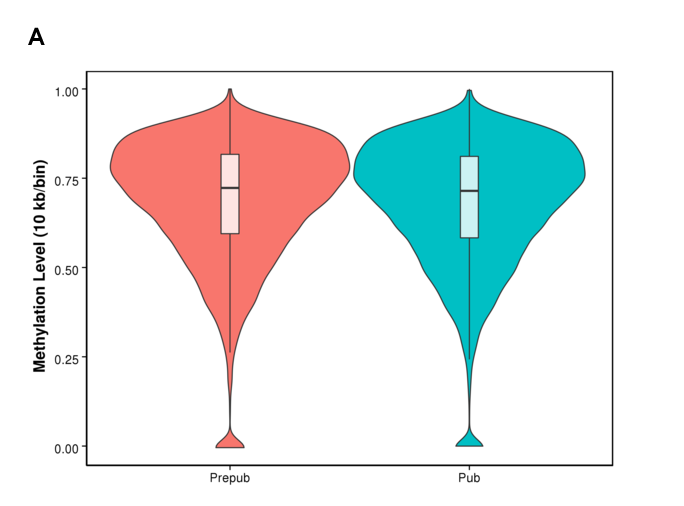


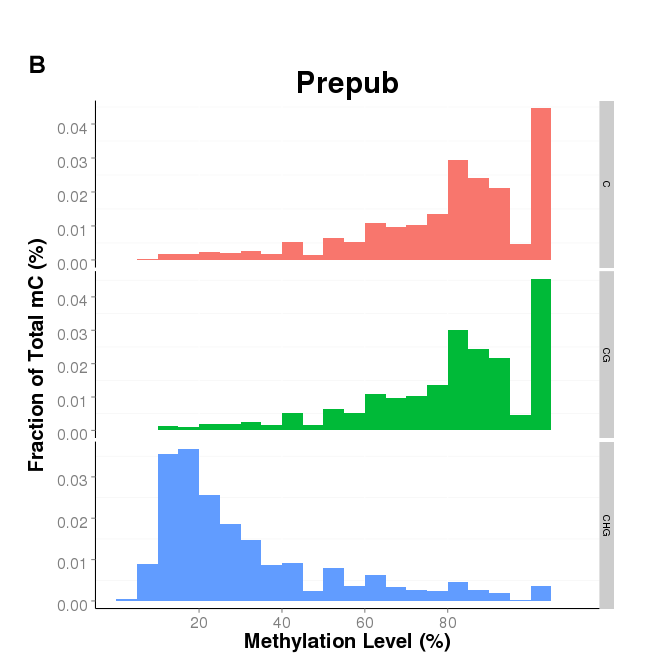


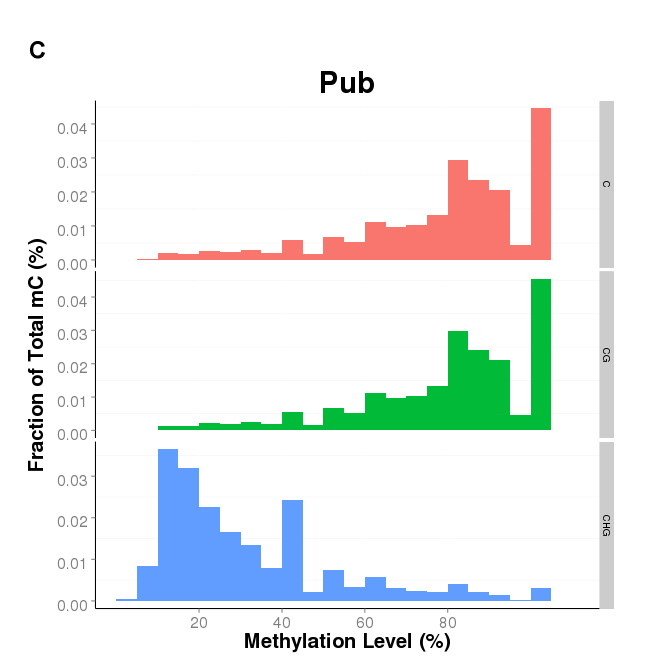


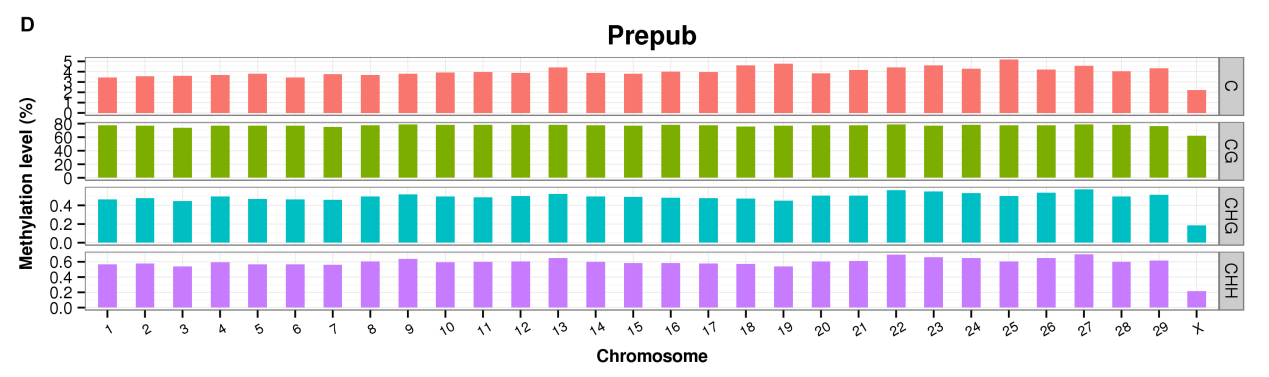


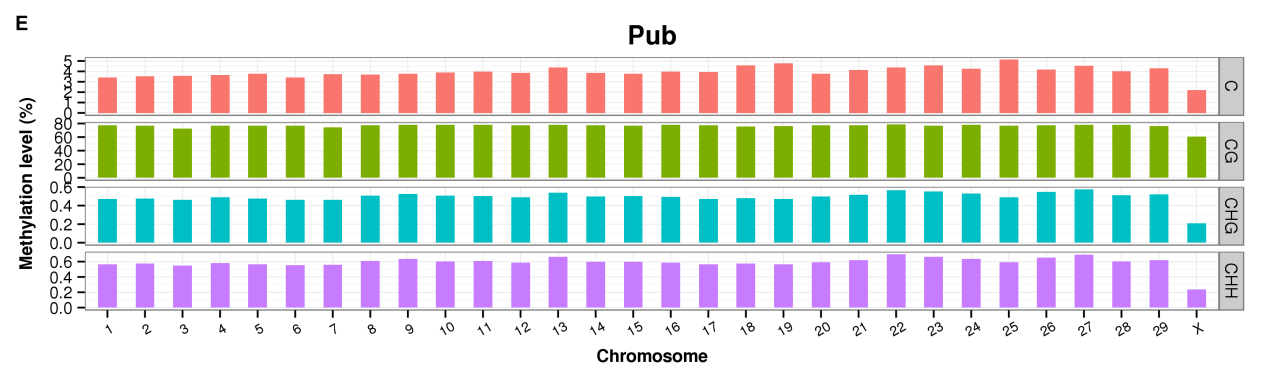

Supplement: S2 Fig — (DOCX) [file pone.0165327.s002.docx]
