# Supplementary material for: DNA Methylation Patterns in the Hypothalamus of Female Pubertal Goats
Source: PLoS One. 2016 Oct 27;11(10):e0165327. doi: 10.1371/journal.pone.0165327 (PMC5082945; doi:10.1371/journal.pone.0165327)
Supplement: S1 Table — (DOCX) [file pone.0165327.s009.docx]

| **Samples** | **mC percent(%)** | **mCpG percent(%)** | **mCHG percent(%)** | **mCHH percent(%)** |
| --- | --- | --- | --- | --- |
| Prepuberty | 1.91% | 38.33% | 0.16% | 0.00% |
| Puberty | 1.87% | 37.43% | 0.17% | 0.00% |

The percentage of different content mC in prepuberty and puberty

The number of different content mC in prepuberty and puberty

| **Samples** | **mC** | **mCG** | **mCHG** | **mCHH** |
| --- | --- | --- | --- | --- |
| Prepuberty | 19102397 (100%) | 18743971 (98.12%) | 358426 (1.88%) | 0 (0.00%) |
| Puberty | 18678817 (100%) | 18304862 (98.00%) | 373955 (2.00%) | 0 (0.00%) |
